# Beyond the Mevalonate Pathway: Control of Post-Prenylation Processing by Mutant p53

**DOI:** 10.3389/fonc.2020.595034

**Published:** 2020-11-05

**Authors:** Carla M Borini Etichetti, Evelyn Arel Zalazar, Nabila Cocordano, Javier Girardini

**Affiliations:** ^1^Instituto de Fisiología Experimental de Rosario, IFISE, CONICET-UNR, Rosario, Argentina; ^2^Instituto de Inmunología Clínica y Experimental de Rosario, IDICER, CONICET-UNR, Rosario, Argentina

**Keywords:** metastasis, carboxymethylation, actin cytoskeleton, CAAX proteins, cancer, methionine restriction, methionine stress

## Abstract

Missense mutations in the *TP53* gene are among the most frequent alterations in human cancer. Consequently, many tumors show high expression of p53 point mutants, which may acquire novel activities that contribute to develop aggressive tumors. An unexpected aspect of mutant p53 function was uncovered by showing that some mutants can increase the malignant phenotype of tumor cells through alteration of the mevalonate pathway. Among metabolites generated through this pathway, isoprenoids are of particular interest, since they participate in a complex process of posttranslational modification known as prenylation. Recent evidence proposes that mutant p53 also enhances this process through transcriptional activation of *ICMT*, the gene encoding the methyl transferase responsible for the last step of protein prenylation. In this way, mutant p53 may act at different levels to promote prenylation of key proteins in tumorigenesis, including several members of the RAS and RHO families. Instead, wild type p53 acts in the opposite way, downregulating mevalonate pathway genes and *ICMT*. This oncogenic circuit also allows to establish potential connections with other metabolic pathways. The demand of acetyl-CoA for the mevalonate pathway may pose limitations in cell metabolism. Likewise, the dependence on S-adenosyl methionine for carboxymethylation, may expose cells to methionine stress. The involvement of protein prenylation in tumor progression offers a novel perspective to understand the antitumoral effects of mevalonate pathway inhibitors, such as statins, and to explore novel therapeutic strategies.

## Introduction

*TP53*, the gene encoding the tumor suppressor p53, is one of the most frequently mutated genes in human cancer (1). More than 70% of *TP53* alterations are missense mutations, leading to the conspicuous presence of p53 point mutants in tumors (2). Mounting evidence has supported the notion that these mutants cooperate with tumorigenesis though the acquisition of novel activities (3). Particularly, animal models provided compelling proof of the ability of p53 point mutants to promote the development of aggressive tumors. Intense research on the mechanisms underlying this effect has revealed a complex scenario (4). Mutant p53 can be considered as a pleiotropic factor that affects cell behavior by altering the function of different interactors. In this context, the presence of specific arrays of interactors combined with patterns of active signaling pathways may explain the manifold activities described for p53 mutants (5). Most mutations are found in the DNA Binding Domain, and a few codons concentrate the highest mutation frequencies. Although the development of aggressive tumors appears as a common biological outcome of most p53 mutants, some differences were also reported (6). The ability to cooperate with oncogenic mechanisms and the exclusive presence in tumor cells make mutant p53 an attractive therapeutic target. Therefore, much effort is concentrated in the study of its function. In this regard, the unexpected finding that mutant p53 alters the expression of mevalonate (MVA) pathway genes (7) opened new avenues to understand the importance of metabolism in tumor cell biology.

## Taking Control of the Mevalonate Pathway, Mutant vs Wild Type p53

**Figure 1:**
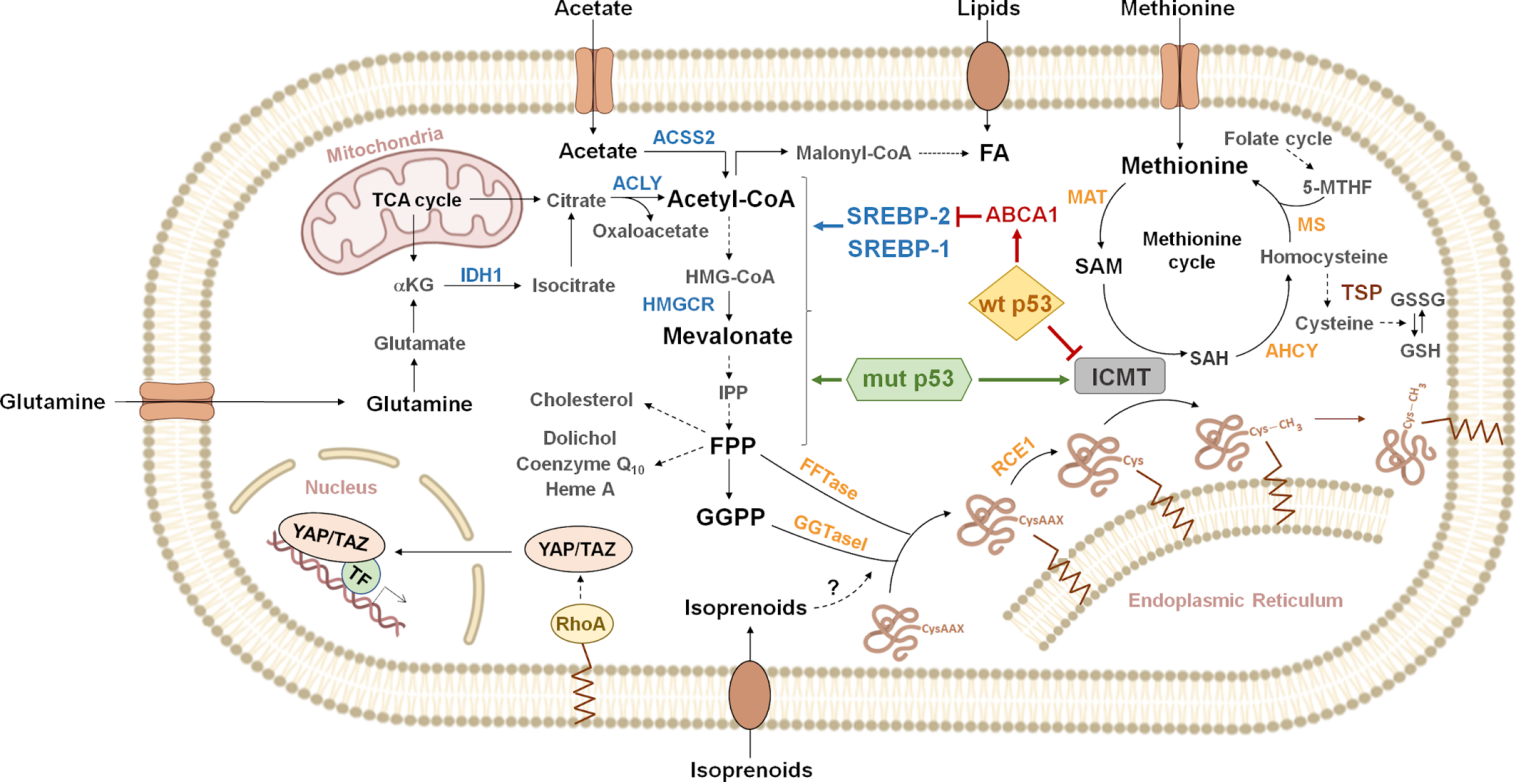
Overview of metabolic pathways connected to protein prenylation. Citrate generated from glutamine or the tricarboxylic acid cycle (TCA cycle) is cleaved by ATP-citrate lyase (ACLY) to acetyl-CoA and oxaloacetate. Acetyl-CoA can also be synthesized by cytoplasmic acetyl-CoA synthetase (ACSS2) from exogenous acetate. Acetyl-CoA, can be carboxylated to malonyl-CoA, to produce fatty acids (FA). Lipids can also be incorporated through exogenous uptake. Alternatively, acetyl-CoA enters the mevalonate pathway, where three molecules are condensed in a two-step reaction to produce 3-hydroxy-3-methylglutaryl CoA (HMG-CoA). HMG-CoA is then reduced by 3-hydroxy-3-methylglutaryl-CoA reductase (HMGCR) to produce mevalonate. Mevalonate is then converted into isopentenyl-diphosphate (IPP) through a series of enzymatic steps. IPP serves as a monomeric unit for the consequent synthesis of farnesyl diphosphate (FPP), geranylgeranyl diphosphate (GGPP) and other downstream metabolites (cholesterol, dolichol, coenzyme Q10, heme A, etc.). The isoprenoid moiety of FPP or GGPP may be covalently attached to cysteine residues on the carboxyl terminus of some proteins, in the first step of the protein prenylation pathway. For example, the activity of RHOA is regulated by geranylgeranylation, which localizes RHOA to the plasma membrane. RHOA promotes the nuclear localization and activity of the Yes-associated protein (YAP) and transcriptional co-activator with PDZ-binding motif (TAZ). Gain of function p53 mutants (mut p53) functionally interact with sterol regulatory element binding proteins (SREBPs) to drive increased expression of mevalonate pathway genes. In contrast, wild type p53 (wt p53) represses the mevalonate pathway genes through inhibition of SREBP-2 maturation, as a consequence of transcriptional induction of ATP binding cassette subfamily A member 1 (*ABCA1)*. Additionally, p53 point mutants can induce isoprenylcysteine carboxymethyltransferase (ICMT) expression, while wt p53 exerts the opposite effect, through transcriptional repression. ICMT catalyzes protein carboxymethylation, the last step of the protein prenylation pathway. The methyl donor in this reaction is S-adenosyl methionine (SAM), which is produced from the essential aminoacid methionine in the rate-limiting reaction catalyzed by methionine adenosyl transferase (MAT). SAM is transformed into S-adenosyl homocysteine (SAH), which can be used to regenerate methionine through the methionine cycle. Homocysteine can be derived to the transsulfuration pathway (TSP) to synthesize glutathione (GSH). Dashed arrows represent multiple enzymatic steps. Indications on reversibility of enzymatic reactions and subcellular localization of some enzymes have been omitted for simplicity. Enzymes known to be regulated by SREBPs are shown in blue. IDH1, isocitrate dehydrogenase 1; α-KG, α-ketoglutarate; FFTase, farnesyl transferase; GGTaseI, geranylgeranyl transferase 1; RCE1, RAS converting enzyme 1; AHCY, adenosylhomocysteinase; MS, methionine synthase; 5-MTHF, 5-methyltetrahydrofolate; GSSG, oxidized glutathione; TF, transcription factors.

The pathological role of alterations on the MVA pathway was initially proposed based on the observation that inhibitors of the enzyme that catalyzes the rate-limiting step (3-hydroxy-3-methyl-glutaryl-CoA reductase, HMGCR), known as statins, reduced the proliferation of tumor cells (8, 9). This pathway allows the biosynthesis of cholesterol and isoprenoids from acetyl-CoA (**Figure 1**). The isoprenoid intermediates farnesyl and geranylgeranyl may be covalently attached to cysteine residues on the carboxyl terminus of proteins, in the first step of the protein prenylation pathway, a complex mechanism of posttranslational modification (10). The connection between mutant p53 and the MVA pathway was unveiled following the observation that several p53 point mutants promoted an aggressive phenotype in three-dimensional (3D) cultures of breast cancer cells (7). The finding that endogenous p53R273H enhanced the expression of at least 17 MVA pathway genes, along with evidence from elegant pharmacologic manipulation of the pathway, led to propose that enhanced flux through the MVA pathway was responsible for the phenotype associated to mutant p53. The expression of MVA pathway genes is under control of Sterol Responsive Element Binding Proteins (SREBPs), which induce transcription in response to low cholesterol levels (11). The recruitment of mutant p53 on the promoters of MVA pathway genes in the vicinity of Sterol Responsive Elements (SREs) as well as the ability of p53R273H to interact with SREBPs suggest that mutant p53 acts as a transcriptional co-activator. Supporting the idea that MVA pathway alteration cooperates with tumor progression, high expression of MVA pathway genes was correlated with poor clinical outcome in breast cancer patients (7).

Protein geranylgeranylation appears to be crucial in the effect of mutant p53. Inhibition of geranylgeranyl transferase I (GGTaseI) attenuated the invasive morphology of MDA-MB-231 cells in 3D cultures, similar to endogenous mutant p53 downregulation. In contrast, inhibition of enzymes that derive the flux of the pathway to other molecules, such as squalene synthase (SQS) and farnesyl transferase (FTase), had no effect. Moreover, addition of geranylgeranyl diphosphate (GGPP) recovered the invasive morphology in cells where mutant p53 was downregulated (7). Furthermore, mutant p53 depletion or HMGCR inhibition by statins reduced the nuclear localization and activity of Yes-Associated Protein (YAP) and Transcriptional coactivator with PDZ-binding motif (TAZ) (12), the transcriptional module of the Hippo pathway, through a mechanism that involves Ras homolog family member A (RHOA) prenylation. Hyperactivation of YAP and TAZ has been increasingly associated to proliferation and metastasis (13). Similarly, YAP/TAZ inactivation was not observed upon inhibition of SQS and FTase, but was phenocopied by GGTaseI inhibition. Moreover, adding GGPP reverted the effect of upstream inhibition of the MVA pathway (12).

The finding that wild type p53 (wt p53) repressed the expression of MVA pathway genes provides strong support to the idea that alteration of this pathway may be a critical event in tumor progression. In this case, an indirect mechanism was described, involving inhibition of SREBP-2 maturation (14). This effect was mediated by the transcriptional induction by wt p53 of ATP binding cassette subfamily A member 1 (*ABCA1)*, which encodes a protein involved in the retrograde transport of cholesterol from the plasma membrane to the endoplasmic reticulum (ER). SREBPs are produced as inactive precursors anchored to the cytosolic side of the ER. Maturation can be stimulated by low cholesterol levels in the ER, which triggers a complex process that leads to proteolytic cleavage and nuclear import of SREBPs (15, 16). Analysis of cancer databases showed that *ABCA1* expression was lower in colon, breast and liver carcinomas comparing with normal tissues. Likewise, *Abca1* inactivation enhanced tumorigenesis in an experimental model of hepatocellular carcinoma (HCC). Additional evidence from animal models strongly supports the notion that ABCA1 is relevant for the tumor suppressive function of wt p53 (14). Noteworthy, wt p53 was also reported to repress the expression of SREBP1-c, suggesting that the interplay between the p53 pathway and SREBPs is even more complex (17, 18).

## Post Prenylation Processing and the p53 Pathway

The posttranslational processing pathway known as prenylation involves three stages (**Figure 1**). First, the addition of farnesyl or geranylgeranyl, to a cysteine residue close to the carboxyl terminus of proteins (19), catalyzed by FTase or GGTaseI, respectively. The prenylated cysteine is typically part of a CAAX motif (C: cysteine; A: aliphatic amino acid; X: any amino acid), although other motifs such as CXC can also be targeted (20). Second, the terminal amino acids following the prenylated cysteine are removed by the specific peptidase RAS Converting Endoprotease 1 (RCE1) in the ER (21). Third, Isoprenylcysteine Carboxyl Methyltransferase (ICMT), also an integral membrane protein of the ER, catalyzes the methylation of the free carboxyl terminus on the cysteine. This modification provides an uncharged hydrophobic carboxyl terminus, which increases protein interaction with biological membranes and/or modifies its ability to interact with other proteins (22). Only one member of the ICMT methyltransferase class is encoded in mammalian genomes and lacks homology to other methyltransferases (23). Interestingly, methylation of prenylated proteins is absent in ICMT^-/-^ cells, which indicates that ICMT is the only enzyme able to catalyze this reaction (24). A connection between the p53 pathway and post-prenylation processing was established by showing that wt and mutant p53 regulate *ICMT* expression (25). Several p53 point mutants induced *ICMT* expression in breast, colon, and lung cancer cell lines. This effect was associated to transcriptional activation, since mutant p53 was recruited on the *ICMT* promoter and was able to enhance its activity. Moreover, promoter activation and enhanced endogenous gene expression were observed in p53 null cells, showing that this activity is a novel function acquired by mutants. In contrast, wt p53 was also found on the *ICMT* promoter but repressed promoter activity and reduced mRNA and protein levels. Interestingly, the effects of wt and mutant p53 were shown to depend on different promoter regions, indicating that they act through different mechanisms. This evidence suggests that the acquisition of missense mutations on *TP53* may exert a strong effect on *ICMT* expression by complementary mechanisms. The repressive function of wt p53 may be lost upon mutation of *TP53*, while the presence of point mutants may enhance gene expression by wt p53-independent mechanisms. Underlining the clinical relevance of the connection between the p53 pathway and post-prenylation processing, *ICMT* expression was found to be significantly reduced in patients classified as wt p53, but was increased in mutant p53 patients (25). The discussed evidence suggests that deregulated expression of ICMT may cooperate with tumor progression. In support to this idea, high ICMT levels were found in hepatocellular carcinoma patients and ICMT overexpression enhanced proliferation and migration in normal liver cells (26). Similarly, ICMT overexpression in H1299 non-small cell lung carcinoma (NSCLC) cells increased clonogenic potential *in vitro* and tumorigenesis in a xenograft model. Moreover, analysis of breast and lung cancer databases showed that high *ICMT* expression was correlated with reduced survival (25).

## ICMT Targets in Oncogenesis

ICMT substrates are distributed among different families (**Table 1**), complicating the rationalization of its pathological effects. In addition to RAS and RHO families of GTPases, more than 200 CAAX proteins have been predicted based on structural analysis (70, 71). Polypeptides ending in CXC, as the doubly geranylgeranylated RAB family, are also modified by this pathway. The identification of RAS family members as ICMT substrates reinforced the notion that protein prenylation may play a role in cancer (72, 73). Deletion of *Icmt* reduced KRAS-induced transformation *in vitro* (74) and neoplastic lesions in a mouse model of myeloproliferative syndrome (75). Moreover, genetic ablation of *ICMT* in RAS-transformed human breast primary cells and human breast cancer cell lines harboring mutant RAS, reduced tumor formation in xenograft models (76). Intriguingly, *Icmt* inactivation in a KRAS-driven mouse model of pancreatic carcinoma increased the number of pancreatic neoplasias and promoted tumor progression (77). Impairment of Notch signaling through deregulation of RAB7 and RAB8 was suggested as responsible for this effect. Considering the impact of mutant p53 as a promoter of pancreatic cancer (78), It will be interesting to explore the interplay between the MVA pathway and protein prenylation in this pathology.

**Table 1:** List of prenylated proteins and ICMT substrates.

Protein Name	CAAX motif	Prenyl Group	ICMT substrate	Reference
G protein-coupled receptor kinase 1 (GRK1)	CLVS	15C	Yes	(27, 28)
G protein subunit gamma transducin 1 (GNGT1/GNG1)	CVIS	15C	Yes	(29, 30)
G protein subunit gamma 2 (GNG2)	CAIL	20C	Yes	(29, 30)
Lamin B1 (LMNB1)	CAIM	15C	Yes	(31–33)
Lamin A (LMNA)	CSIM	15C	Yes	(34, 35)
ERAS	CSVA	15C	Yes	(36)
HRAS	CVLS	15C	Yes	(37, 38)
KRAS4A	CIIM	15C	Yes	(24, 37, 38)
KRAS4B	CVIM	15C	Yes	(24, 37, 38)
NRAS	CVVM	15C	Yes	(37, 38)
RAB3B	CSC	20C	Yes	(39)
RAB3D	CSC	20C	Yes	(39)
RAB4A	CGC	20C	Yes	(40, 41)
RAB6A	CSC	20C	Yes	(39)
RAB7A	CSC	20C	Yes	(41)
RAB8A	CVLL	20C	Yes	(41, 42)
RAB13	CSLG	20C	Yes	(41, 42)
RAB18	CSVL	20C	Yes	(41)
RAB23	CSVP	20C	Yes	(41)
RAB27A	CGC	20C	Yes	(41)
RALA	CCIL	20C	Yes	(43–45)
RALB	CCLL	20C	Yes	(44, 45)
RHEB1	CHLM	15C	Yes	(36, 46)
RHEB2	CSVM	15C	Yes	(36, 46)
RHOA	CLVL	20C	Yes	(47, 48)
RHOB	CKVL	15C/20C *	Yes	(49–51)
RHOC	CPIL	20C	Yes	(49, 51)
RHOD	CVVT	15C	Yes	(49)
RHOH	CKIF	15C/20C *	Yes	(49)
CDC42	CCIF	20C	Yes	(37, 52)
RAC1	CLLL	20C	Yes	(53, 54)
RAC2	CSLL	20C	Yes	(37, 43, 55)
RAC3	CTVF	20C	Yes	(54, 56)
Phosphodiesterase 6A (PDE6A/PDEα)	CCIQ	15C	Yes	(28, 57, 58)
Phosphodiesterase 6B (PDE6B/PDEβ)	CCIL	20C	Yes	(28, 57, 58)
Lamin B2 (LMNB2)	CYVM	15C	Methylation	(33, 59)
RAB3A	CAC	20C	Methylation	(20, 60)
RAP1A	CLLL	20C	Methylation	(61)
RAP1B	CQLL	20C	Methylation	(62)
STK11/LKB1	CKQQ	15C	ND	(63)
PTP4A1/PTPCAAX1	CCIQ	15C/20C*	ND	(64)
PTP4A2/PTPCAAX2	CCVQ	15C/20C*	ND	(64)
RAP2A	CNIQ	15C	ND	(65)
RAP2B	CVIL	20C	ND	(65)
RAP2C	CVVQ	20C	ND	(66, 67)
PPP1R16B/TIMAP	CRIS	15C	ND	(68, 69)

The specific CAAX or CXC motifs and the type of isoprenoid (15C farnesyl group; 20C, geranyl-geranyl group), are indicated. Cases where there is experimental evidence on the involvement of ICMT are indicated. Methylation: proteins shown to be carboxymethylated but without evidence on the involvement of ICMT. ND, not determined. (*) Proteins reported to be farnesylated or geranyl-geranylated.

The deregulated action of ICMT on RHO GTPases may promote invasiveness and metastasis through alteration of cytoskeleton remodelling and cell motility. Accordingly, ICMT inhibition reduced migration and invasion in MDA-MB-231 cells (53), concomitant with decreased RHOA and RAC1 activity. The ability of miR-100 to attenuate lamellipodia formation, matrix metallopeptidase 2 (MMP2) activation and metastasis in hepatocellular carcinoma cells was associated to ICMT-RAC1 signaling inhibition (79). Likewise, reduced migration, invasion and metastasis were observed in HT-1080 fibrosarcoma cells upon ICMT inhibition (80), which was associated to RAB4A impaired function. ICMT overexpression in H1299 cells significantly affected actin cytoskeleton, suggesting an effect on RHO GTPases (25). Interestingly, some evidences reported differential effects of ICMT on subcellular localization and/or expression levels of protein substrates, arguing for a role in the concerted regulation of prenylated proteins. For example, ICMT inhibition reduced RHOA half-life, but enhanced RAS stability (74, 81). Lack of ICMT had different effects on the subcellular localization of RAS and RHO family members (49), and on the localization and stability of RALA and RALB. Dynamic regulation of protein carboxymethylation may have relevant consequences as suggested by the identification of carboxylesterase 1 (CES1), a carboxylesterase affecting the methylation status of RHOA. Interestingly, RHOA activity and cytoskeleton organization in breast cancer cells were similarly affected by *CES1* silencing and ICMT overexpression (82).

## Acetyl-CoA and Metabolic Stress in Tumor Cells

Availability of acetyl-CoA may be a critical aspect in tumor cells that sustain aggressive phenotypes by exploiting the MVA pathway. Acetyl-CoA is the starting point of the MVA pathway; however, it is also required for other important pathways, as fatty acids (FA) biosynthesis (**Figure 1**). An important source of acetyl-CoA is citrate produced in the mitochondria by the tricarboxylic acid (TCA) cycle, which can be converted in the cytosol into oxaloacetate and acetyl-CoA by ATP citrate-lyase (ACLY) (83). In addition, exogenous acetate may be directly converted into acetyl-CoA by cytoplasmic acetyl-CoA synthetase (ACSS2) (84). Glutamine uptake also allows the indirect production of acetyl-CoA through a series of reactions that take place in the cytosol (85, 86). A strong requirement of acetyl-CoA may expose tumor cells to the dependence on specific metabolic capabilities, forcing cells to shape their metabolism. Accordingly, there is evidence showing enhanced activity of ACLY (87) and ACSS2 (88) in cancer cells, as well as of isocitrate dehydrogenase 1 (IDH1) (89, 90), which catalyzes reductive carboxylation in the conversion of glutamine into acetyl-CoA. Expression of these genes is regulated by SREBPs suggesting the intriguing possibility that they may be induced by mutant p53 and repressed by wt p53 (91–95). Oxygen availability is frequently limited in the tumor microenvironment and nutrient uptake is highly conditioned by the degree of neovascularization (96). Entry of pyruvate into the mitochondria may be inhibited under hypoxic conditions (97), downregulating the TCA cycle and citrate production. Under these conditions, acetate and glutamine as alternative sources of acetyl-CoA may become critical. Moreover, if uptake of exogenous lipids is not able to satisfy the high demand in proliferating cells, active FA biosynthesis may be expected to compete with the MVA pathway for acetyl-CoA. In this scenario, strategies aimed at interfering with alternative acetyl-CoA sources may be effective to counteract cancer cell proliferation.

## ICMT Links the Mevalonate Pathway With Methionine Metabolism

The methyl donor in protein carboxymethylation is S-adenosyl methionine (SAM), which is produced from the essential aminoacid methionine, in a reaction catalyzed by methionine adenosyl transferase (MAT). SAM is also the methyl donor in other reactions, including methylation of DNA, RNA, non-prenylated proteins and in polyamine biosynthesis. Upon methylation, SAM is transformed into S-adenosyl homocysteine (SAH), which can be used to regenerate methionine through the methionine cycle (98) (**Figure 1**). This cycle is closely interconnected with two other metabolic processes. Hydrolysis of SAH, catalyzed by adenosylhomocysteinase (AHCY), produces homocysteine, which can react with 5-methyl-tetra-hydrofolate (5-MTH) generated in the folate cycle, giving back methionine. Alternatively, homocysteine can be diverted to the transsulfuration pathway that ultimately leads to the synthesis of glutathione (GSH). Alteration of methionine cycle enzymes were related to cancer. For example, *MAT2A* and *MAT2B*, the genes coding for the subunits of the most abundant MAT isoenzyme, were found upregulated in tumors and cancer-initiating cells (99, 100). The close connection between SAM and the one-carbon metabolic network suggests that cell context and nutritional state may affect ICMT activity. Methionine availability may decrease SAM levels, thereby limiting ICMT catalyzed carboxymethylation. Therefore, limiting methionine uptake may have a selective inhibitory effect on cancer cells that benefit from ICMT hyperactivation. Accordingly, pioneering observations reported a marked requirement of methionine on transformed rat and human cells (101). Moreover, dietary methionine restriction reduced tumor growth and metastasis in animal models, and increased sensitivity to chemotherapeutic agents (98). Nevertheless, the molecular mechanisms underlying these effects are not yet clear.

Homocysteine is a key molecule in the one-carbon network, since it connects the methionine cycle with the folate cycle and GSH production. Under strong oxidative stress conditions, high availability of GSH may be required and, therefore, homocysteine may be preferentially driven to the transsulfuration pathway, precluding the possibility to regenerate methionine. Therefore, enhanced ICMT activity in cells under oxidative stress may further increase the dependency on methionine. Moreover, SAH acts as a negative feedback inhibitor of ICMT (102). Treatment with the AHCY inhibitor adenosine dihaldeyde (AdOx) produced accumulation of SAH (103) and reduced *in vitro* invasion and migration of cancer cell lines (104).

## Discussion

Several metabolites produced by the MVA pathway may affect cell behavior, however, the positive effect of mutant p53 on the expression of MVA pathway genes and *ICMT* underline the relevance of isoprenoids in cancer. Conversely, the negative regulation exerted by wt p53 on SREBP-2 maturation and *ICMT* expression indicates that MVA pathway and carboxymethylation of prenylated proteins should be strictly regulated under physiological conditions. The concerted effects of mutant p53 on MVA and prenylation pathways allow tumor cells to connect both pathways, thereby fostering full modification of prenylated proteins playing key roles in oncogenesis. Still, selective alteration of each pathway may be enough to promote tumor progression. In this way, mutant p53 may activate alternative mechanisms useful to promote tumorigenesis in different contexts. Since exogenous isoprenoids may be incorporated into cancer cells and phosphorylated (105), the intriguing possibility that protein prenylation may be exploited by tumors independently from the MVA pathway may also be considered. Noteworthy, exogenous supplementation of geranylgeraniol counteracted the antitumoral effect of pitavastatin in a xenograft model of ovarian cancer cells (106). The correlation of ICMT expression with clinical outcome and the pro-oncogenic effects observed in experimental systems point at ICMT overexpression as a relevant event in tumor progression. Consequently, the potential of ICMT as a therapeutic target encouraged the identification of inhibitors. Isoprenylated cysteine analogs inhibited ICMT activity and showed antiproliferative effects, however, their mechanism of action is not clear since some of them act as modulators of RAS chaperones (107, 108). Indole-based molecules were also proposed, such as Cysmethynil (109), a competitive inhibitor with respect to isoprenylated cysteine and a non-competitive inhibitor with respect to SAM, which showed antitumor activity *in vitro* and *in vivo* (10, 110, 111). In summary, alteration of MVA pathway and protein prenylation by mutant p53 revealed interesting connections to explore. Understanding the role of less studied ICMT substrates in cancer and the study of mechanisms that regulate ICMT activity will be critical to dissect the molecular mechanisms underlying ICMT pathological effects.

## Author Contributions

JG designed and wrote the manuscript. CBE contributed to manuscript writing and performed the figures. EAZ and NC contributed to the manuscript writing and figures. All authors contributed to the article and approved the submitted version.

## Funding

This work was supported by grants to JG from Instituto Nacional del Cáncer (INC, Asistencia Financiera III) and from Agencia Nacional de Promoción Científica y Tecnológica (ANPCyT. PICT CABBIO 4758). CBE is a postdoctoral fellow from the National Council for Scientific and Technological Research of Argentina (CONICET). EAZ and NC are doctoral fellows from the National Council for Scientific and Technological Research of Argentina (CONICET).

## Conflict of Interest

The authors declare that the research was conducted in the absence of any commercial or financial relationships that could be construed as a potential conflict of interest.
